# Development and validation of a predictive model for assessing the risk of follicular carcinoma in thyroid nodules identified as suspicious by intraoperative frozen section

**DOI:** 10.3389/fendo.2024.1431247

**Published:** 2024-09-26

**Authors:** Cheng Li, Yong Luo, Yongli Gan, Yan Jiang, Qi Li, Jin Huang

**Affiliations:** ^1^ Department of Thyroid Surgery, Ningbo Medical Center Lihuili Hospital, Ningbo, China; ^2^ Ningbo Clinical Diagnostic Pathology Center, Ningbo, China; ^3^ Department of Ultrasound, Ningbo Medical Center Lihuili Hospital, Ningbo, China; ^4^ Department of Surgery, The Second Hospital of Ninghai County, Ningbo, China

**Keywords:** predictive modeling, intraoperative frozen section, thyroid nodules, follicular carcinoma, risk assessment, ultrasound features, nomogram

## Abstract

**Introduction:**

Follicular thyroid carcinoma (FTC) is the second most common thyroid malignancy and is characterized by a higher risk of distant metastasis compared to papillary thyroid cancer. Intraoperative frozen section (IOFS) diagnosis of FTC is challenging due to its limited sensitivity and accuracy, leading to uncertainty in intraoperative surgical decision-making. In response, we developed a predictive model to assess the risk of follicular carcinoma in thyroid nodules identified as suspicious for follicular neoplasm by IOFS.

**Methods:**

This model was derived from preoperative clinical and ultrasound data of 493 patients who underwent thyroid surgery at Ningbo Medical Center Lihuili Hospital. It identified five significant predictors of follicular carcinoma: nodule size, thyroglobulin (Tg) level, hypoechogenicity, lobulated or irregular margins, and thick halo.

**Results:**

The model demonstrated robust discrimination and calibration, with an area under the curve (AUC) of 0.83 (95% CI: 0.77-0.90) in the training set and 0.78 (95% CI: 0.68-0.88) in the validation set. In addition, it achieved a sensitivity of 81.63% (95% CI: 69.39-91.84) and 68.00% (95% CI: 48.00--4.00), a specificity of 77.42% (95% CI: 72.18-82.66) and 72.51% (95% CI: 65.50-78.96), an accuracy of 78.1% (95% CI: 73.4-82.4) and 71.9% (95% CI: 65.3-78.6), a positive predictive value (PPV) of 41. 67% (95% CI: 35.65-48.84) and 26.79% (95% CI: 19.40-34.33), respectively, and a negative predictive value (NPV) of 95.61% (95% CI: 92.86-97.99) and 94.07% (95% CI: 90.44-97.08) in the training and validation sets, respectively.

**Conclusion:**

The model can accurately rule out FTC in low-risk nodules, thereby providing surgeons with a practical tool to determine the necessary extent of surgical intervention for nodules flagged as suspicious by IOFS.

## 1 Introduction

Thyroid cancer is the most common endocrine malignancy worldwide, with an estimated 586,202 new cases and 43,646 deaths in 2020, making it the ninth most common cancer worldwide (1). Follicular thyroid carcinoma (FTC), which accounts for 10-15% of all thyroid cancers (2), is the second most common type after papillary thyroid carcinoma (PTC). FTC is associated with a significantly higher risk of distant metastasis than PTC, with metastasis rates ranging from 19% to 33.7% in FTC patients—significantly higher than the 2.3% to 7% observed in PTC patients (3–8). In response to this risk, the American Thyroid Association (ATA) guidelines recommend total thyroidectomy for widely invasive FTC or minimally invasive FTC with high-risk factors (9). This surgical strategy not only allows for radioactive iodine ablation of potential residual disease postoperatively but also improves detection of recurrence by facilitating monitoring of postoperative thyroglobulin (Tg) levels, a marker for differentiated thyroid carcinoma, thus reducing the likelihood of recurrence (10). Therefore, accurate preoperative and intraoperative diagnosis of FTC is critical to guide surgical decision-making and determine the extent of surgical intervention required.

However, the preoperative and intraoperative diagnosis of FTC remains challenging. A definitive pathologic diagnosis of FTC requires evidence of vascular or capsular invasion, typically confirmed by postoperative paraffin pathology (11). As a result, preoperative fine needle aspiration (FNA) cytology is inadequate for the diagnosis of FTC and often yields indeterminate results, such as the Bethesda System for Reporting Thyroid Cytopathology (TBSRTC) categories III or IV (12). For nodules with indeterminate FNA results, intraoperative frozen section (IOFS) can help differentiate the malignant from benign lesions and guide surgical decision-making (13, 14). However, the sensitivity of IOFS for the diagnosis of FTC is also limited, with only 3.4% to 10.5% of cases being accurately identified (15, 16); Most of the remainder are initially reported as suspicious of follicular neoplasm (17), requiring definitive diagnosis by paraffin pathology postoperatively. This diagnostic uncertainty may result in some patients returning to surgery for contralateral thyroidectomy after a diagnosis of FTC, while others may have undergone total thyroidectomy only to later discover that the nodule was benign, such as a follicular adenoma.

In response to this diagnostic challenge, researchers have been actively developing methods to predict FTC. Various studies have linked specific clinical characteristics (18) and ultrasound features (19–22) to the likelihood of FTC. In addition, predictive models have been developed to distinguish FTC from follicular adenoma in thyroid nodules diagnosed by postoperative paraffin pathology (23–25). Despite these advances, there is currently no model designed to assess the risk of FTC intraoperatively in nodules identified as suspicious for follicular neoplasm by IOFS.

Therefore, this study aims to develop an intraoperative predictive model to assess the risk of FTC in thyroid nodules identified as suspicious for follicular neoplasm by IOFS. The model will utilize preoperative clinical and ultrasound data to improve the accuracy of intraoperative diagnoses, particularly in accurately ruling out FTC in low-risk nodules. This enhancement will aid surgical decision-making and guide the selection of appropriate surgical interventions.

## 2 Methods

### 2.1 Ethical approval and study design

This retrospective cohort study was approved by the Ethics Committee of Ningbo Medical Center Lihuili Hospital (approval number: KY2024SL082-01). The study adhered to the Transparent Reporting of a Multivariable Prediction Model for Individual Prognosis or Diagnosis (TRIPOD) statement for model development, validation, and reporting (26). The study included patients who underwent thyroid surgery at Ningbo Medical Center Lihuili Hospital between January 1, 2019 and December 31, 2023. The hospital has two separate campuses: the Eastern Campus, located on the eastern side of the city, and the Xingning Campus, located in the center of the city. Each campus has independent laboratories, ultrasound facilities, operating rooms, and medical teams. Both campuses utilize the same Hospital Information System (HIS). All pathology diagnostic services, including cytology, molecular biology, IOFS, and postoperative paraffin pathology, are provided by the Ningbo Clinical Pathology Diagnostic Center in a uniform manner.

The diagnosis and treatment of thyroid nodules at the hospital follow the guidelines of ATA (9). Patients with thyroid nodules underwent an initial evaluation, including physical examination, thyroid function tests, and ultrasound. Nodules classified as Thyroid Imaging Reporting and Data System (TI-RADS) (27) category 4A or higher on ultrasound were recommended for FNA to facilitate cytologic examination. In addition to FNA Bethesda VI results and BRAF V600E mutation, our institution’s surgical indications for nodules without definitive preoperative malignancy evidence (such as those included in this study) include Bethesda categories IV or V, persistent Bethesda III after repeated FNA, compressive symptoms, cosmetic concerns, rapid growth, or other clinical symptoms necessitating surgery. Before surgery, all patients were tested for Tg and thyroglobulin antibody (TgAb) levels. IOFS was routinely performed during surgery to guide the surgical approach for nodules with indeterminate FNA results or those that had not undergone FNA.

### 2.2 Study cohort establishment and dataset partitioning

The study cohort was established by enrolling patients who met the following criteria. Inclusion criteria were (all conditions had to be met): 1) Patients underwent surgery for FNA TBSRTC IV or V, or repeated FNA results of TBSRTC III, or nodules causing compressive symptoms, cosmetic concerns, rapid growth, or other clinical symptoms necessitating surgery; 2) IOFS of the thyroid nodule is consistent with a follicular cell-derived neoplasm; 3) IOFS of the nodule indicates suspicion of a follicular neoplasm, but without definitive evidence of vascular or capsular invasion, requiring postoperative paraffin pathology for definitive diagnosis; 4) IOFS cannot diagnose the nodule as another type of malignancy. Exclusion criteria were (any of the following): 1) incomplete clinical or imaging data; 2) history of partial or total thyroid surgery; 3) presence of another nodule diagnosed as malignant by FNA or IOFS, or detection of BRAF V600E mutation by preoperative molecular testing, in the same or contralateral lobe; 4) nodules with inconclusive pathologic type by postoperative paraffin pathology; 5) preoperative confirmation of central (level VI) or lateral neck (levels II-V) lymph node metastasis; 6) preoperative confirmation of distant metastasis of thyroid cancer.

Patients meeting these criteria were identified from the hospital’s HIS to form the study cohort. Patients admitted to the Eastern Campus were assigned to the training set, while those admitted to the Xingning Campus were assigned to the validation set.

### 2.3 Candidate predictor selection and data collection

A comprehensive literature review identified several clinical and ultrasound features as potential predictors of FTC. These included age (19, 28), gender (19), smoking status (28), serum Tg levels (16, 29, 30), and serum TgAb levels (31). Thyroid nodule size (18, 19, 32), along with ultrasound features such as echogenicity (19–21, 23, 24, 33), margin (20, 23, 33, 34), halo (19, 21, 23, 24), and calcification (18, 20, 21, 23, 35, 36) were also relevant. Additional ultrasound descriptors from the TI-RADS, including nodule composition and shape, were employed in this analysis.

At our institution, preoperative serum Tg and TgAb levels were measured by immunochemiluminescent assay using the Siemens ADVIA Centaur XP Immunoassay System. The reference ranges are 0.973 – 29.58 *µ*g/L for Tg and 0 – 60.0 IU/mL for TgAb. Accordingly, cut-off values for Tg and TgAb in this study were set at 29.58 *µ*g/L and 60.0 IU/mL, respectively. Test results below these cut-offs were considered “negative,” and those above were considered “positive.” Nodule size was determined by measuring the maximum diameter on ultrasound. Ultrasound features were graded according to the TI-RADS criteria. A halo, defined as a thin rim of decreased echogenicity surrounding the nodule (19), was categorized by thickness as absent, thin (< 2 mm), or thick (≥ 2 mm) (37).

Data on these predictors were collected from the hospital’s HIS and recorded in a Microsoft ACCESS database. To minimize the potential for human error, data entry was performed by two researchers and automatically checked for discrepancies by ACCESS. For ultrasound features, one researcher entered data based on diagnostic reports, while another sonographer, blinded to the patient’s final diagnosis, entered data based on the original images. Any discrepancies were resolved by a senior sonographer. IOFS and paraffin pathology data were obtained based on reports from the hospital pathology database. Any ambiguous cases were resolved by a senior pathologist.

### 2.4 Model construction and visualization

The model was constructed using the training set. The dependent variable was the paraffin pathology diagnosis of the nodule, which was classified into two categories: FTC and non-FTC (pathological types other than FTC). The independent variables were the candidate predictors identified in the literature review. To select the predictors for the model, we employed the Least Absolute Shrinkage and Selection Operator (LASSO) regression (38), which is particularly useful when the number of predictors is large relative to the number of observations (39). LASSO is a regression analysis method that performs variable selection by shrinking the coefficients of less influential predictors to zero via increasing the value of a penalty parameter, λ. The optimal value of λ was selected based on the one-standard-error (1-SE) criterion (40). This criterion identifies the optimal λ as the largest value that places the LASSO’s binomial deviance, a measure of model fit calculated using 10-fold cross-validation, within 1-SE of the minimum binomial deviance. The predictors with non-zero coefficients at this optimal λ were identified as the final predictors. A logistic regression model was then constructed on the training set using these predictors. The optimal threshold for the model was determined as the value that maximized the Youden index in the training set. To facilitate the clinical application of the model, we developed a nomogram based on the logistic regression model.

### 2.5 Model validation

The model was validated on both the training and validation sets. The discrimination of the model was assessed using the receiver operating characteristic (ROC) curve and the area under the curve (AUC). Calibration of the model was assessed using the calibration curve, which plots the predicted probabilities against the observed incidence of FTC. A perfectly calibrated model would lie on the diagonal reference line. The clinical utility of the model was determined by decision curve analysis (DCA), which calculates the net benefit by comparing the model’s performance against the treat-all strategy (assuming all nodules are FTC) and the treat-none strategy (assuming none of the nodules are FTC) (41). If the model provides a higher net benefit than both strategies over a range of threshold probabilities, it is considered clinically useful. The diagnostic performance of the model was evaluated by calculating sensitivity, specificity, accuracy, positive predictive value (PPV), and negative predictive value (NPV) at the optimal threshold in both the training and validation sets.

### 2.6 Statistical analysis

All modeling procedures were performed using the R software (42) (https://www.R-project.org/), version 4.3.3. the LASSO regression was performed using the package “glmnet” (43). The logistic regression model and nomogram were constructed using the package “rms” (44). The ROC curve, calibration curve, and DCA were generated using the packages “pROC” (45), “probably” (46), and “rmda” (47), respectively. Plots were generated using the package “ggplot2” (48). Continuous variables were presented as mean ± standard deviation (SD) and compared using the t-test. Categorical variables were presented as frequencies and percentages and compared using the chi-squared test or Fisher’s exact test. A p-value of less than 0.05 was considered statistically significant.

## 3 Results

**Figure 1:**
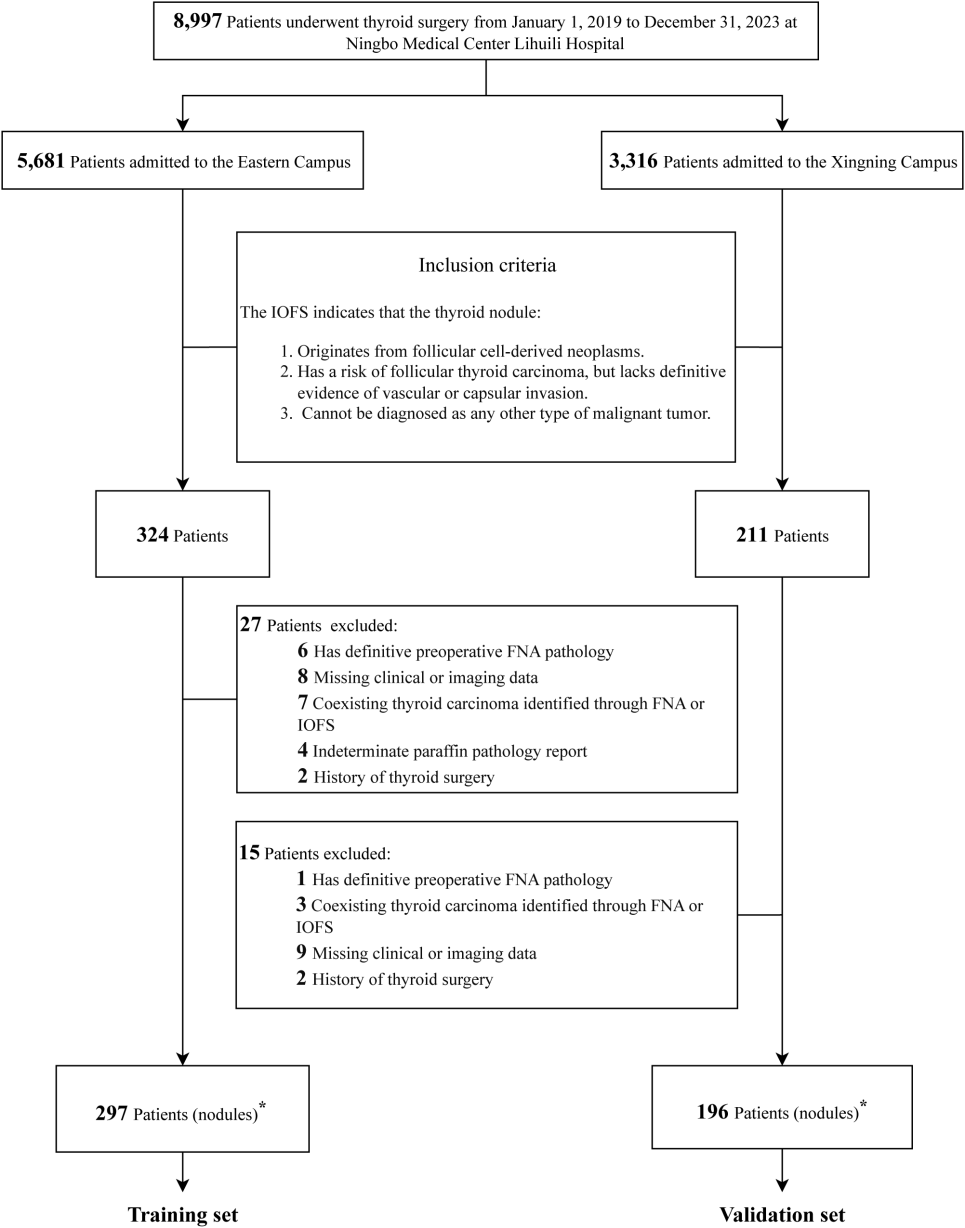
Flowchart of study cohort establishment and dataset partitioning. IOFS, intraoperative frozen section; FNA, fine-needle aspiration. ^∗^The number of thyroid nodules is equal to the number of patients.

A total of 8,997 patients who underwent thyroid surgery at Ningbo Medical Center Lihuili Hospital between January 1, 2019, and December 31, 2023, were identified from the hospital’s HIS. Of these patients, 5,681 were treated at the Eastern Campus and 3,316 at the Xingning Campus. A total of 493 patients met the inclusion and exclusion criteria and were included in the study cohort. Of these, 297 patients from the Eastern Campus were designated as the training set, while 196 patients from the Xingning Campus were designated as the validation set, as detailed in **Figure 1**.

**Table 1** presents the baseline characteristics of the training and validation sets. In the training set (n = 297), 49 patients (16.5%) were diagnosed with FTC, while 248 (83.5%) had non-FTC diagnoses. The non-FTC group comprised 8 (2.7%) with PTC, 95 (32.0%) with follicular tumor of uncertain malignant potential, 34 (11.4%) with follicular adenoma, 107 (36.0%) with follicular nodular disease, and 4 (1.3%) with other benign types. The validation set (n = 196) included 25 patients (12.8%) with FTC and 171 (87.2%) with non-FTC diagnoses, consisting of 10 (5.1%) with PTC, 72 (36.7%) with follicular tumor of uncertain malignant potential, 12 (6.1%) with follicular adenoma, 76 (38.8%) with follicular nodular disease, and 1 (0.5%) with other benign types.

**Table 1:** Baseline characteristics and comparison of data distribution across training and validation sets.

	All N=493	Training set N=297	Testing set N=196	*p*-value
Pathology type^a^, No. (%)				0.13
Follicular carcinoma	74 (15.0)	49 (16.5)	25 (12.8)	
Papillary carcinoma	18 (3.7)	8 (2.7)	10 (5.1)	
Follicular tumor of uncertain malignant potential	167 (33.9)	95 (32.0)	72 (36.7)	
Follicular adenoma	46 (9.3)	34 (11.4)	12 (6.1)	
Follicular nodular disease	183 (37.1)	107 (36.0)	76 (38.8)	
Other benign types	5 (1.0)	4 (1.3)	1 (0.5)	
Dependent variable, No. (%)				0.31
Follicular carcinoma	74 (15.0)	49 (16.5)	25 (12.8)	
Non-follicular carcinoma^b^	419 (85.0)	248 (83.5)	171 (87.2)	
Gender, No. (%)				0.47
Female	352 (71.4)	208 (70.0)	144 (73.5)	
Male	141 (28.6)	89 (30.0)	52 (26.5)	
Age, mean(SD), years	50.8 (13.8)	50.0 (13.9)	52.0 (13.7)	0.13
Current smoking, No. (%)				0.83
No	432 (87.6)	259 (87.2)	173 (88.3)	
Yes	61 (12.4)	38 (12.8)	23 (11.7)	
Nodule size, mean(SD), mm	36.1 (15.4)	37.0 (16.1)	34.7 (14.1)	0.09
Tg test result^c^, n(%)				0.92
Negative	332 (67.3)	199 (67.0)	133 (67.9)	
Positive	161 (32.7)	98 (33.0)	63 (32.1)	
TgAb test result^d^, No. (%)				0.81
Negative	431 (87.4)	261 (87.9)	170 (86.7)	
Positive	62 (12.6)	36 (12.1)	26 (13.3)	
US nodule composition, No. (%)				0.14
Mixed cystic and solid	63 (12.8)	38 (12.8)	25 (12.8)	
Solid or almost completely solid	416 (84.4)	247 (83.2)	169 (86.2)	
Spongiform	14 (2.8)	12 (4.0)	2 (1.0)	
US nodule shape, No. (%)				0.17
Taller than wide	15 (3.0)	6 (2.0)	9 (4.6)	
Wider than tall	478 (97.0)	291 (98.0)	187 (95.4)	
US nodule echogenicity, No. (%)				0.32
Isoechoic	81 (16.4)	45 (15.2)	36 (18.4)	
Hyperechoic	40 (8.1)	21 (7.1)	19 (9.7)	
Hypoechoic	372 (75.5)	231 (77.8)	141 (71.9)	
US nodule margin, No. (%)				0.90
Smooth	444 (90.1)	266 (89.6)	178 (90.8)	
Ill-defined	19 (3.9)	12 (4.0)	7 (3.6)	
Lobulated or irregular	30 (6.1)	19 (6.4)	11 (5.6)	
US calcification: large comet-tail artifacts^e^, No. (%)				0.41
Absent	487 (98.8)	292 (98.3)	195 (99.5)	
Present	6 (1.2)	5 (1.7)	1 (0.5)	
US calcification: macrocalcifications^f^, No. (%)				0.91
Absent	438 (88.8)	263 (88.6)	175 (89.3)	
Present	55 (11.2)	34 (11.4)	21 (10.7)	
US calcification: punctate echogenic foci^g^, No. (%)				1.00
Absent	463 (93.9)	279 (93.9)	184 (93.9)	
Present	30 (6.1)	18 (6.1)	12 (6.1)	
US nodule halo feature, No. (%)				0.61
Thin	172 (34.9)	106 (35.7)	66 (33.7)	
Thick	124 (25.2)	70 (23.6)	54 (27.6)	
Absence	197 (40.0)	121 (40.7)	76 (38.8)	

US, ultrasound. Tg, thyroglobulin; TgAb, thyroglobulin antibody; SD, standard deviation.

^a^All patients underwent surgery because of fine-needle aspiration (FNA) results of Bethesda System for Reporting Thyroid Cytopathology (TBSRTC) category IV or V, or repeated FNA results of TBSRTC category III, or nodules causing compressive symptoms, cosmetic concerns, rapid growth, or other clinical symptoms necessitating surgery.

^b^Non-follicular carcinoma includes all pathology types that do not meet the criteria for follicular thyroid carcinoma. In this study, it includes papillary carcinoma, follicular tumor of uncertain malignant potential, follicular adenoma, follicular nodular disease, and other benign types.

^c,d^The cut-off values for Tg and TgAb in this study were set at 29.58 µg/L and 60.0 IU/mL, respectively. Test results below these cut-offs were considered “negative,” and those above were considered “positive”.

^e,f,g^The three types of calcifications are defined according to the ultrasound Thyroid Imaging Reporting and Data System(TI-RADS) (27). This table lists three different calcification features separately because it is possible for these three types of calcifications to coexist and therefore require separate reporting and scoring.

Females constituted 71.4% of the total cohort (352/493 patients), with similar proportions in the training (70.0%, 208/297) and validation (73.5%, 144/196) sets. The mean age of the entire cohort was 50.8 ± 13.8 years, with comparable ages in the training (50.0 ± 13.9 years) and validation (52.0 ± 13.7 years) sets. Non-smokers accounted for 87.6% of the cohort (432/493 patients), with similar distributions in the training (86.5%, 257/297) and validation (89.3%, 175/196) sets. The mean nodule size was 36.1 ± 15.4 mm overall, with slight variations between the training (37.0 ± 16.1 mm) and validation (34.7 ± 14.1 mm) sets.

Additional clinical characteristics, including Tg and TgAb test results, and ultrasound features such as nodule composition, shape, echogenicity, margin, and calcification characteristics, are detailed in **Table 1**. All baseline characteristics were comparable between the training and validation sets, with no significant differences observed (all p > 0.05), thus ensuring a reliable assessment of the model’s performance and its potential generalizability to new patient populations.

**Figure 2:**
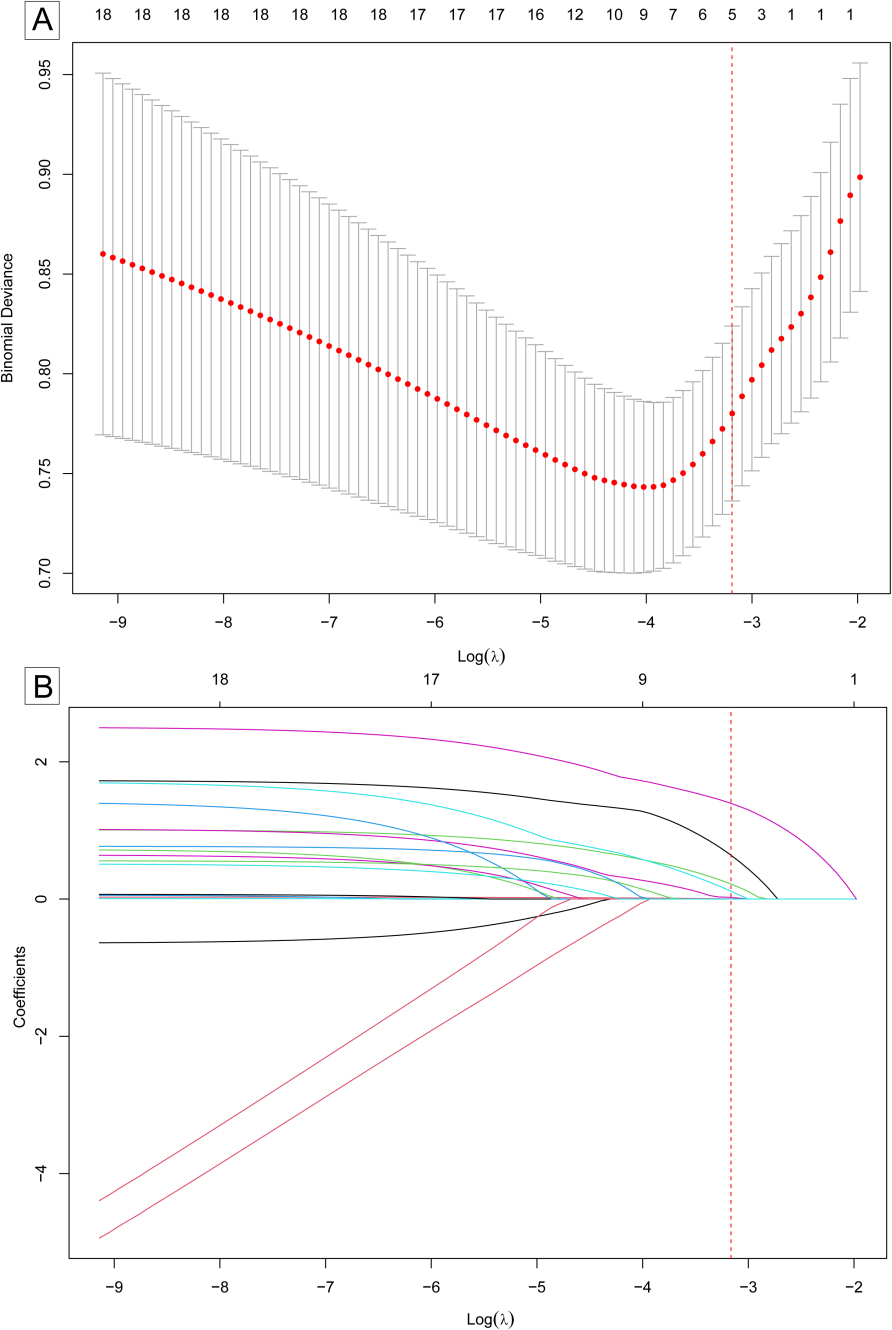
Selection of predictors for follicular thyroid carcinoma risk model using LASSO regression. **(A)** Optimal value of λ selection by 10-fold cross-validation. The “Binomial deviance” on the y-axis is a measure of the goodness of fit of the model. The optimal value of λ was determined by the one-standard-error (1-SE) of the minimum binomial deviance (marked by the red dotted vertical line). **(B)** The LASSO coefficients were shrunk by the increasing value of λ. At the optimal value of λ(marked by the red dotted vertical line), five variables with non-zero coefficients remained. The five variables were selected as predictors for the model. LASSO, least absolute shrinkage and selection operator. SE, standard error.

Univariate analysis in the training set revealed six predictors significantly associated with FTC: nodule size, Tg levels, smoking status, and ultrasound features of echogenicity, margin, and halo (all p < 0.05). These results are detailed in **Table 2**. The LASSO regression identified the optimal value of λ based on the 1-SE criterion, as illustrated in **Figure 2A**. At the optimal value of λ, five coefficients of the predictors remained non-zero: nodule size, Tg levels, and ultrasound features of echogenicity, margin, and halo, as illustrated in **Figure 2B**. A logistic regression model constructed using these predictors in the training set demonstrated a good fit, with a χ^2^ value of 67.69 (p < 0.01) and a pseudo-R^2^ of 0.34. The model demonstrated that larger nodule size (odds ratio (OR): 1.03, 95% confidence interval (CI): 1.01-1.06), positive Tg levels (OR: 2.47, 95% CI: 1.18-5.16), hypoechogenicity (OR: 4.90, 95% CI: 1.01-23.69), lobulated or irregular margins (OR: 7. 70, 95% CI: 2.23-26.54) and thick halo (OR: 13.84, 95% CI: 4.73-40.53) significantly increased the risk of FTC compared to their respective reference categories (all p < 0.05), as detailed in **Table 3**.

**Table 2:** Univariate analysis of potential predictors for follicular carcinoma in the training set.

	All N=297	Non-follicular carcinoma^a^N=248	Follicular carcinomaN=49	*p*-value
Gender, No. (%)				0.78
Female	208 (70.0)	175 (70.6)	33 (67.3)	
Male	89 (30.0)	73 (29.4)	16 (32.7)	
Age, mean(SD), years	50.0 (13.9)	50.4 (13.5)	48.4 (15.8)	0.42
Current smoking, No. (%)				0.01
No	259 (87.2)	222 (89.5)	37 (75.5)	
Yes	38 (12.8)	26 (10.5)	12 (24.5)	
Nodule size, mean(SD), mm	37.0 (16.1)	36.0 (15.0)	42.2 (20.3)	0.047^*^
Tg test result^b^, No. (%)				0.02
Negative	199 (67.0)	174 (70.2)	25 (51.0)	
Positive	98 (33.0)	74 (29.8)	24 (49.0)	
TgAb test result^c^, No. (%)				0.79
Negative	261 (87.9)	219 (88.3)	42 (85.7)	
Positive	36 (12.1)	29 (11.7)	7 (14.3)	
US nodule composition, No. (%)				0.21
Mixed cystic and solid	38 (12.8)	33 (13.3)	5 (10.2)	
Solid or almost completely solid	247 (83.2)	203 (81.9)	44 (89.8)	
Spongiform	12 (4.0)	12 (4.8)	0 (0.0)	
US nodule shape, No. (%)				1.00
Taller than wide	6 (2.0)	5 (2.0)	1 (2.0)	
Wider than tall	291 (98.0)	243 (98.0)	48 (98.0)	
US nodule echogenicity, No. (%)				0.03^*^
Isoechoic	45 (15.2)	43 (17.3)	2 (4.1)	
Hyperechoic	21 (7.1)	19 (7.7)	2 (4.1)	
Hypoechoic	231 (77.8)	186 (75.0)	45 (91.8)	
US nodule margin, No. (%)				0.01
Smooth	266 (89.6)	227 (91.5)	39 (79.6)	
Ill-defined	12 (4.0)	10 (4.0)	2 (4.1)	
Lobulated or irregular	19 (6.4)	11 (4.4)	8 (16.3)	
US calcification: large comet-tail artifacts^d^, No. (%)			0.60	
Absent	292 (98.3)	243 (98.0)	49 (100.0)	
Present	5 (1.7)	5 (2.0)	0 (0.0)	
US calcification: macrocalcifications^e^, No. (%)				0.35
Absent	263 (88.6)	222 (89.5)	41 (83.7)	
Present	34 (11.4)	26 (10.5)	8 (16.3)	
US calcification: punctate echogenic foci^f^, No. (%)				0.52
Absent	279 (93.9)	234 (94.4)	45 (91.8)	
Present	18 (6.1)	14 (5.6)	4 (8.2)	
US nodule halo feature, No. (%)				<0.01^**^
Thin	106 (35.7)	101 (40.7)	5 (10.2)	
Thick	70 (23.6)	41 (16.5)	29 (59.2)	
Absence	121 (40.7)	106 (42.7)	15 (30.6)	

US, ultrasound. Tg, thyroglobulin; TgAb, thyroglobulin antibody; SD, standard deviation.

^a^The definition of non-follicular carcinoma are presented in the footnote^a^ of **Table 1**.

^b,c^The definitions of Tg and TgAb as either “positive” or “negative” are Presented in the footnote^b,c^ of **Table 1**.

^d,e,f^See footnote^d,e,f^ in **Table 1**.

^*^ *p* <0.05, ** *p* <0.01.

**Table 3:** Logistic regression model for the prediction of follicular thyroid carcinoma.

Variable	Coefficients(B)			OR		*p*-value
	Estimate	SE	95% CI	Estimate	95% CI	
Intercept	-6.08	1.06	-8.17 to -4.00	0.00	0.00 to 0.02	<0.01^**^
Nodule size	0.03	0.01	0.01 to 0.05	1.03	1.01 to 1.06	0.01^*^
Tg test result^a^						
Negative	0(Reference)			1(Reference)		
Positive	0.91	0.38	0.17 to 1.64	2.47	1.18 to 5.16	0.02^*^
US nodule echogenicity						
Isoechoic	0(Reference)			1(Reference)		
Hyperechoic	1.22	1.11	-0.97 to 3.4	3.37	0.38 to 30.00	0.28
Hypoechoic	1.59	0.80	0.01 to 3.17	4.90	1.01 to 23.69	0.048
US nodule margin						
Smooth	0(Reference)			1(Reference)		
Ill-defined	0.99	0.91	-0.78 to 2.77	2.70	0.46 to 16.00	0.27
Lobulated or irregular	2.04	0.63	0.8 to 3.28	7.70	2.23 to 26.54	<0.01^**^
US nodule halo feature						
Thin	0(Reference)			1(Reference)		
Thick	2.63	0.55	1.55 to 3.7	13.84	4.73 to 40.53	<0.01^**^
Absence	0.73	0.56	-0.37 to 1.83	2.07	0.69 to 6.23	0.19

χ^2^ (8) = 67.69, p <0.01. Pseudo-R^2^ (Cragg-Uhler) = 0.34, akaike information criterion (AIC) = 216.32, bayesian information criterion (BIC) = 249.57.

OR, odds ratio; CI, confidence interval; SE, standard error; Tg, thyroglobulin; US, ultrasound.

a The definitions of Tg as either “positive” or “negative” are Presented in the footnote^*b*^ of **Table 1**.

^*^ *p* <0.05, ** *p* <0.01.

**Figure 3:**
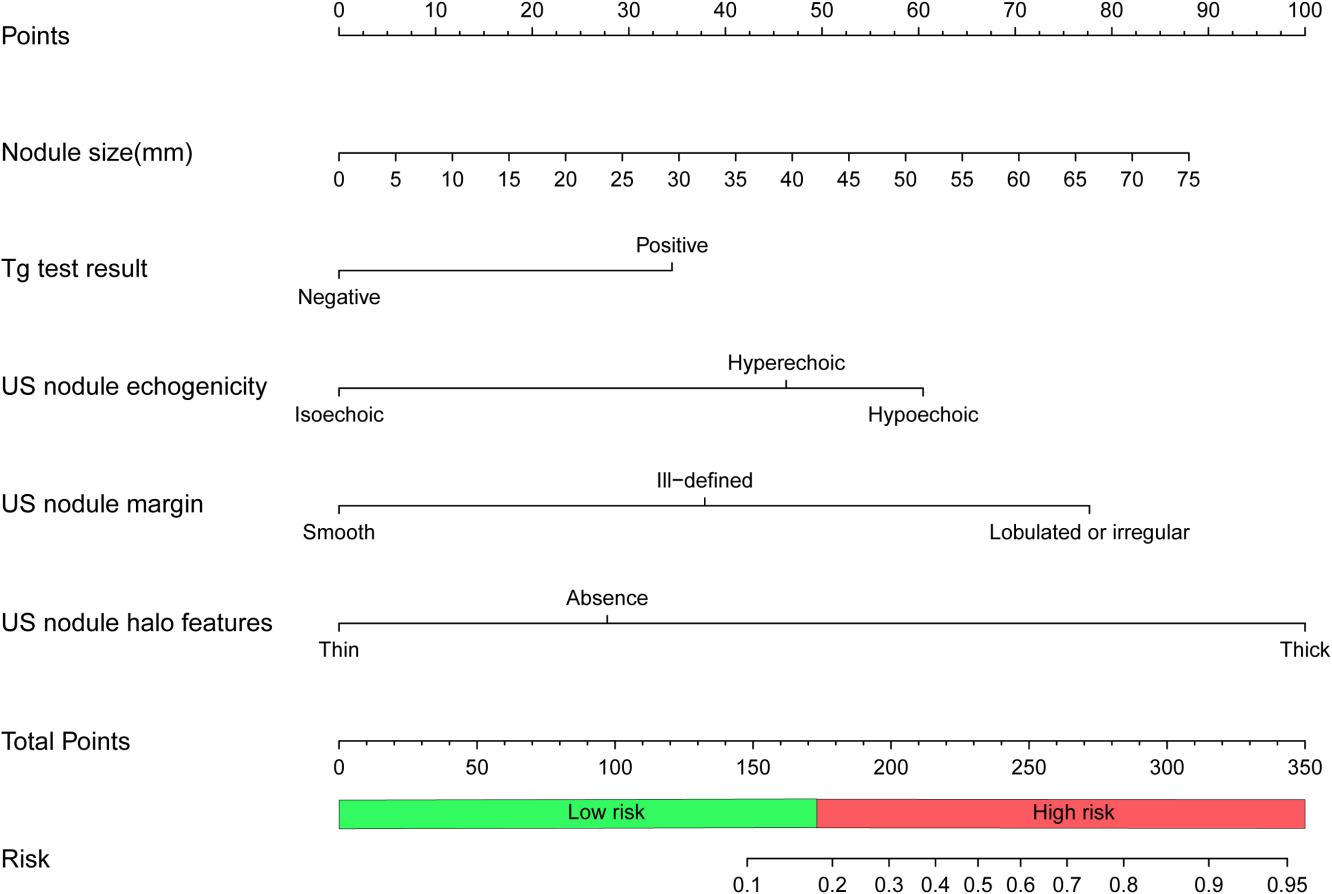
Nomogram to predict follicular thyroid carcinoma risk. To calculate the risk of follicular thyroid carcinoma, locate the features of the nodule on the corresponding axes, then draw a vertical line from each location to the “Points” axis to determine the points for that feature. Add the points of all the features and place the total on the “Total Points” axis, which corresponds to the risk probability of follicular thyroid carcinoma on the “Risk” axis. A total of 173 points(risk probability of 0.175) or more indicates a high risk of follicular thyroid carcinoma. Tg, thyroglobulin; US, ultrasound.

A nomogram was developed based on this logistic regression model, as shown in **Figure 3**. In the training set, the maximum Youden index was 0.59, corresponding to a model probability of 0.175 and a nomogram score of 173 points, which served as the optimal threshold. Consequently, nodules with a nomogram score of 173 points or above were classified as high-risk for FTC, while those with scores below this threshold were classified as low-risk.

**Figure 4:**
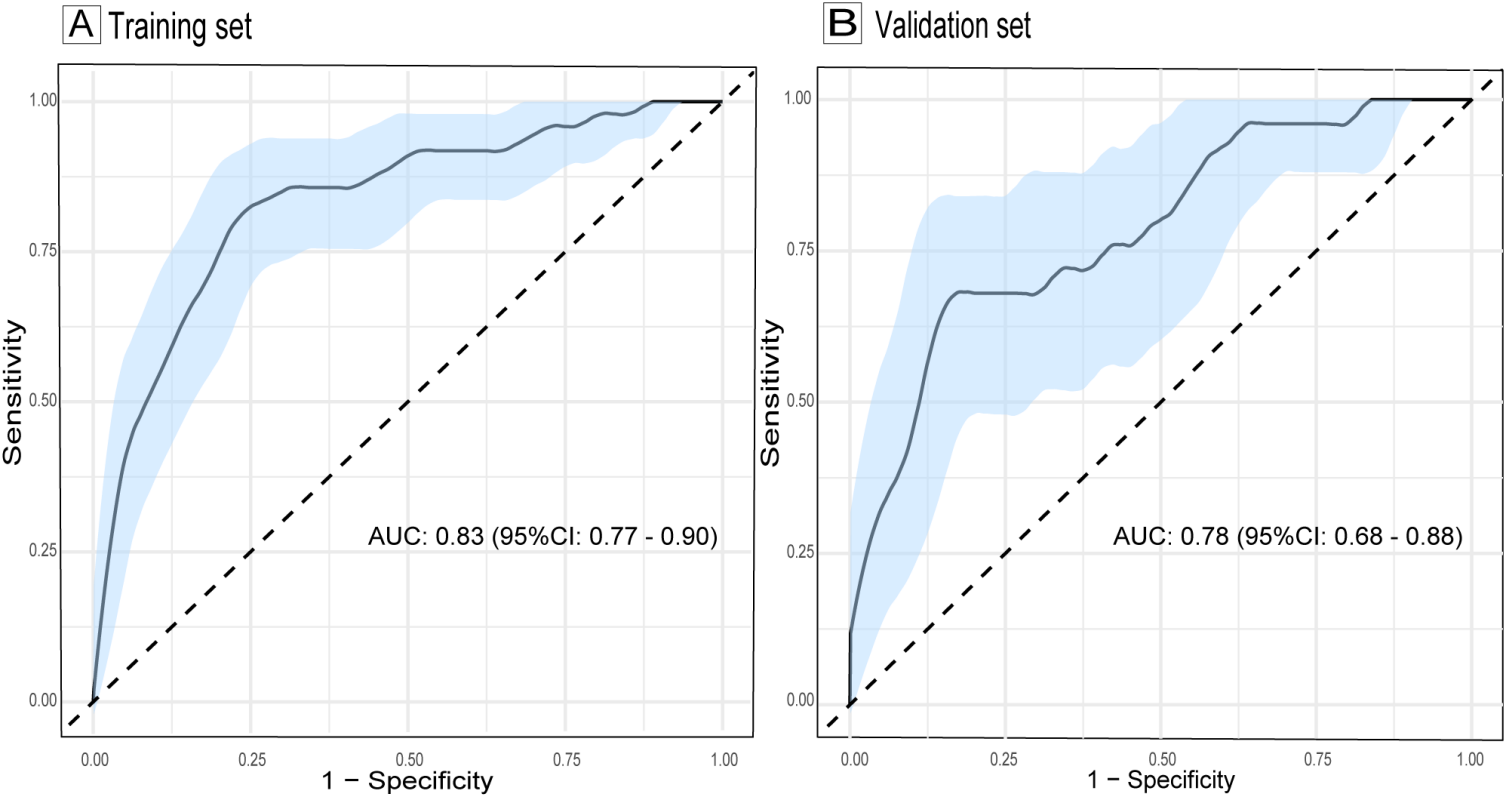
ROC curves evaluating the discrimination of the follicular thyroid carcinoma risk model in training **(A)** and validation sets **(B)**. The light blue shaded area represents the 95% confidence interval of the ROC curves. AUC, area under the curve; ROC, receiver operating characteristic; CI, confidence interval.

**Figure 5:**
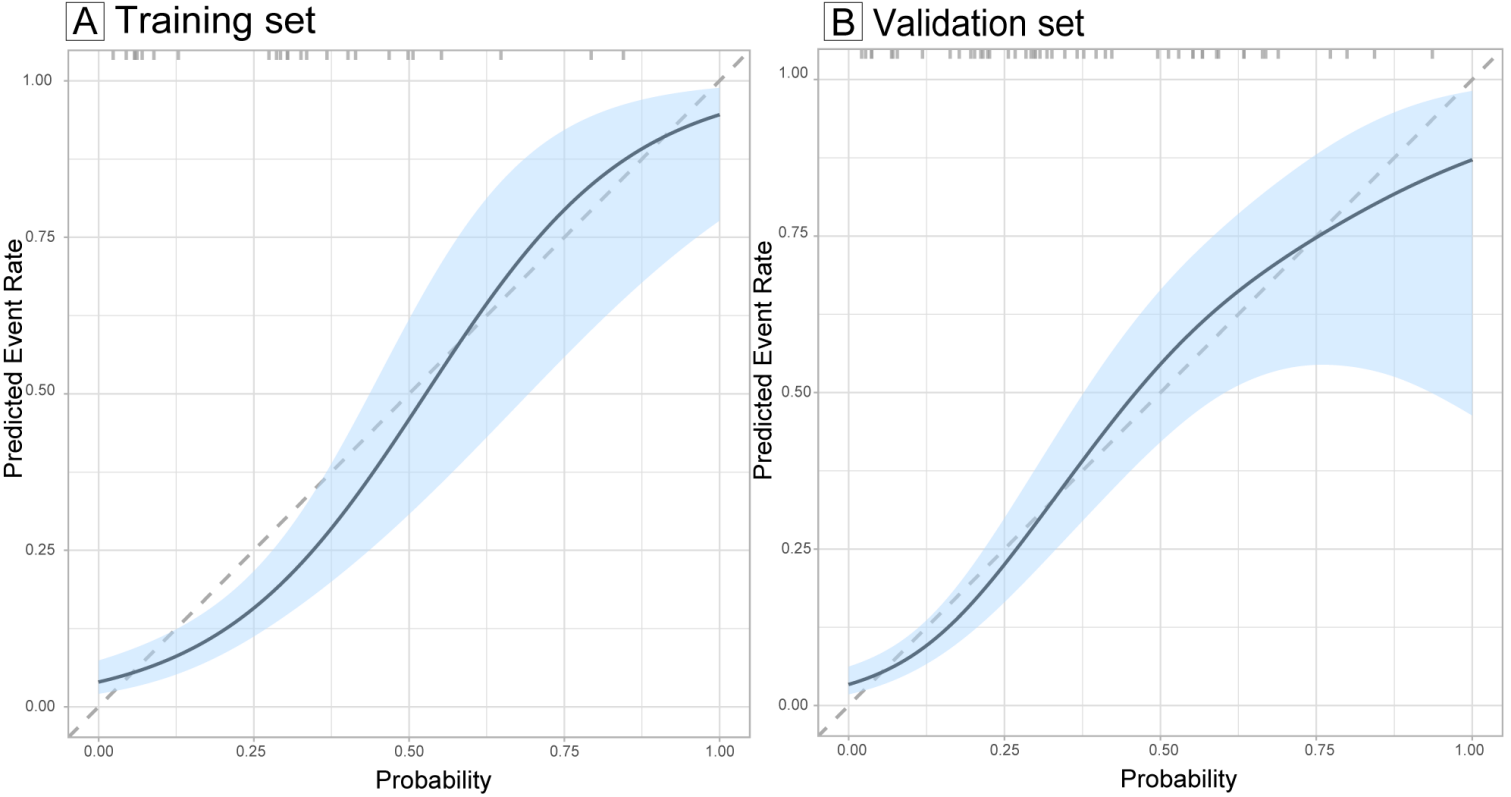
Calibration curves evaluating the calibration of the follicular thyroid carcinoma risk model in training **(A)** and validation sets **(B)**. The calibration curves illustrate the concordance between the predicted probabilities and the actual observed incidence of follicular thyroid carcinoma in the training and validation sets. The diagonal dashed lines serve as a benchmark for perfect prediction. The light blue shaded area represents the 95% confidence interval (CI) of the calibration curves.

**Figure 6:**
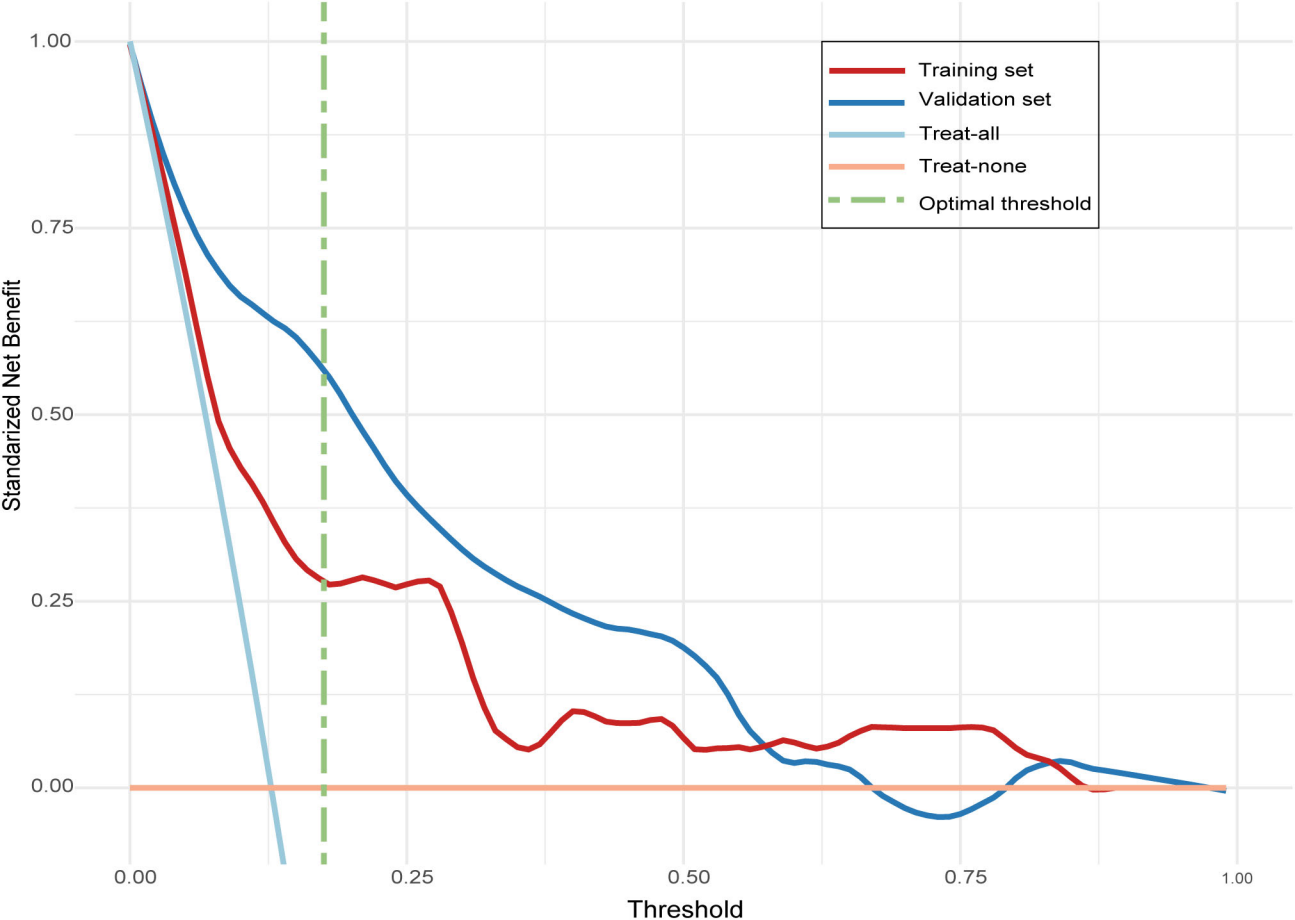
Decision curve analysis (DCA) evaluating the clinical utility of the follicular thyroid carcinoma risk model in training and validation sets. The line labeled “treat-all” represents the net benefit of assuming that all nodules are follicular thyroid carcinoma, and the line labeled “treat-none” represents the net benefit of assuming that no nodules are follicular thyroid carcinoma.

The ROC curve of the model, depicted in **Figure 4**, demonstrated satisfactory discrimination, with an AUC of 0.83 (95% CI: 0.77-0.90) in the training set and 0.78 (95% CI: 0.68-0.88) in the validation set. The calibration curves, depicted in **Figure 5**, demonstrated a satisfactory agreement between the predicted probabilities and the observed incidences of FTC in both sets, indicating an accurate calibration. The DCA, illustrated in **Figure 6**, revealed that the model provided a higher net benefit than both the treat-all and treat-none strategies over a wide range of threshold probabilities, underscoring its clinical utility in both the training and validation sets.

At the optimal threshold, the model achieved a sensitivity of 81.63% (95% CI: 69.39–91.84), a specificity of 77.42% (95% CI: 72.18-82.66), an accuracy of 78.1% (95% CI: 73.4 – 82.4), a PPV of 41.67% (95% CI: 35.65–48.84), and an NPV of 95.61% (95% CI: 92.86–97.99) in the training set. In the validation set, the model achieved a sensitivity of 68.00% (95% CI: 48.00–84.00), a specificity of 72.51% (95% CI: 65.50–78.96), an accuracy of 71.9% (95% CI: 65.3 – 78.6), a PPV of 26.79% (95% CI: 19.40–34.33), and an NPV of 94.07% (95% CI: 90.44-97.08), as detailed in **Table 4**.

**Table 4:** Performance of the predictive model for predicting follicular carcinoma in training and validation sets *^a^*.

Actual Predicted	Follicular carcinomaNo. (%)	Non-follicular carcinoma^b^No. (%)	Sensitivity, %		Specificity, %		Accuracy, %		PPV, %		NPV, %	
			Mean	95%CI	Mean	95%CI	Mean	95%CI	Mean	95%CI	Mean	95%CI
Training set			81.63	69.39 to 91.84	77.42	72.18 to 82.66	78.1	73.4 to 82.8	41.67	35.65 to 48.84	95.61	92.86 to 97.99
High risk	40(81.6)	56(20.0)										
Low risk	9(18.4)	192(80.0)										
Validation set			68.00	48.00 to 84.00	72.51	65.50 to 78.96	71.9	65.3 to 78.6	26.79	19.40 to 34.33	94.07	90.44 to 97.08
High risk	17(68.0)	47(27.5)										
Low risk	8(32.0)	124(72.5)										

PPV, positive predictive value; NPV, negative predictive value; CI, confident interval.

a A predicted nomogram score of 173 points was used as the cutoff value to define high and low risk of follicular carcinoma. A nomogram score ≥ 173 points was defined as “high risk,” and nomogram score <173 points was defined as “low risk”.

b The definition of non-follicular carcinoma are presented in the footnote^*a*^ of **Table 1**.

## 4 Discussion

In this study, we developed an intraoperative predictive model to assess the risk of FTC in thyroid nodules identified by IOFS as suspicious for follicular neoplasm. The model exhibited a high NPV, with 95.61% in the training set and 94.07% in the validation set. This enables surgeons to confidently rule out FTC in low-risk nodules. This level of diagnostic confidence encourages surgeons to adopt more conservative surgical approaches, such as preferring lobectomy over total thyroidectomy. This reduces the number of unnecessary surgeries and the associated risks, including hypoparathyroidism and recurrent laryngeal nerve injury. Ultimately, this enhances patient outcomes and quality of life.

Furthermore, the model has demonstrated strong discrimination, calibration, and clinical utility in both the training and validation sets. This underscores its potential for integration into clinical practice. The predictors incorporated in the model, including nodule size, Tg levels, and ultrasound features such as echogenicity, margin, and halo, are commonly available in clinical settings, facilitating their routine use. The accompanying nomogram serves as a practical tool, aiding surgeons in making informed decisions regarding the necessary extent of surgical intervention.

To our knowledge, this is the first model specifically designed to predict the risk of FTC in this setting. Existing models, such as the one by Yu et al. (16), aimed to predict FTC in all patients undergoing thyroid surgery, but showed a low PPV of 11.1% due to the low prevalence of FTC in unselected thyroid surgery patients (only 61 out of 3,588 cases in the study). Another model by Macias et al. (28) was designed to predict malignancy in nodules with a cytologic diagnosis of follicular neoplasm (Bethesda IV) on FNA. While a significant proportion of these nodules was malignant (33.8% of 151 patients, of which 29.4% were FTC), this model was not exclusively focused on identifying FTC. Furthermore, it did not include cases of FTC that were misclassified as Bethesda I, III, or V due to FNA sampling error or cytologic limitations, which often require further confirmation by IOFS. Therefore, our model addresses a critical need by providing a targeted approach to predict the risk of FTC in nodules during intraoperative examination.

Predictors identified in the model are consistent with previous studies. Larger nodule size, positive Tg levels, ultrasound features of hypoechogenicity, lobulated or irregular margin, and thick halo have been reported as predictors of FTC in several studies (16, 18, 19, 21, 21, 23, 24, 29, 30, 32–34). However, other predictors such as older age (19), male sex (19), the presence of calcifications (20, 23, 35) or microcalcifications (21, 36) on ultrasound, did not show a significant association in our analysis. Furthermore, although smoking showed a significant association in our univariate analysis, it was not included in the final model. This discrepancy may be due to the unique characteristics of our study population or the limited sample size. Further studies with larger cohorts are needed to validate these findings.

Despite its strengths, this study has several limitations. First, due to the study design, some malignancies other than FTC may have been inadvertently included in the non-FTC group. Despite the application of strict inclusion and exclusion criteria, a total of 18 (3.7%) patients diagnosed with PTC were included in this group, mainly because some patients in this study did not undergo preoperative FNA or BRAF V600E mutation testing. This inclusion may affect the performance and generalizability of the model. However, with the increasing use of these diagnostic tools in clinical practice, we anticipate that the impact of this limitation will be minimized. Second, the model showed a relatively low PPV of 41.67% (95% CI: 35.65-48.84) in the training set and 26.79% (95% CI: 19.40-34.33) in the validation set, indicating a high false-positive rate. However, the high NPV of the model indicates that it is more effective in excluding FTC than in confirming it. This still represents a significant clinical benefit. Third, the retrospective nature of the study may introduce selection bias, especially since some patients who did not undergo IOFS were not included in the study. Furthermore, the relatively small number of subjects and an even smaller number of FTC cases compared to the number of predictors could lead to overfitting of the model, although LASSO regression was used to mitigate this risk. Finally, although the model was validated using an independent dataset from another campus of the hospital, it may still be subject to institutional bias and may not generalize well to other populations, underscoring the need for further multicenter studies to validate the model’s performance in different clinical settings.

## Data Availability

The raw data supporting the conclusions of this article will be made available by the authors, without undue reservation.
